# Middle Meningeal Artery Embolization as Standalone Therapy for Chronic Subdural Hematoma with Radiological Herniation Features: A Case Report

**DOI:** 10.3390/neurolint18030052

**Published:** 2026-03-05

**Authors:** Gamaliel Wibowo Soetanto, Elvan Wiyarta

**Affiliations:** 1 Department of Neurology, St Borromeus Hospital, Bandung 40132, Indonesia; 2 Intensive Care Unit, University of Indonesia Hospital, Depok 16424, Indonesia

**Keywords:** hematoma, embolization, endovascular, herniation, primary

## Abstract

**Background:** Chronic subdural hematoma is commonly managed with surgical evacuation when significant mass effect or herniation features are present. Although middle meningeal artery embolization has emerged as an effective adjunctive therapy, evidence supporting its use as standalone treatment in patients with radiological herniation remains limited. **Case Presentation:** We report a 51-year-old man who presented with a three-week history of progressive headache, intermittent confusion, and mild left-sided weakness. Magnetic resonance imaging demonstrated a right-sided chronic subdural hematoma with marked cortical compression and subfalcine herniation. Despite radiological severity, the patient remained neurologically stable. After multidisciplinary discussion, middle meningeal artery embolization was performed as sole therapy via right radial access using a liquid embolic agent. Selective angiography demonstrated pathological neovascular supply from the right middle meningeal artery, which was completely obliterated following embolization without procedural complications. The post-procedural course was uneventful, with progressive clinical improvement. Follow-up non-contrast computed tomography at eight months demonstrated near-complete resolution of the hematoma with normalization of midline structures, and no surgical evacuation was required. **Conclusions:** Standalone middle meningeal artery embolization may represent a feasible therapeutic option in carefully selected clinically stable patients with chronic subdural hematoma and radiological herniation features, though further studies are required to define optimal selection criteria and long-term outcomes.

## 1. Introduction

Chronic subdural hematoma is a frequent neurosurgical condition and a common cause of progressive neurological symptoms in adults, traditionally managed with surgical evacuation when there is significant mass effect or neurological deficit [1]. Despite the effectiveness of burr hole evacuation and subdural drainage, recurrence rates remain substantial, prompting growing interest in therapeutic strategies that address the underlying pathophysiology of the disease rather than mechanical decompression alone [2]. Increasing evidence suggests that chronic subdural hematoma is maintained by a vascularized outer membrane supplied predominantly by branches of the middle meningeal artery, characterized by fragile neovascular networks, inflammatory mediators, and recurrent microhemorrhage [2]. This biological framework has provided the rationale for middle meningeal artery embolization as a disease modifying treatment targeting the source of hematoma persistence and recurrence, a concept increasingly supported in contemporary consensus statements and clinical studies such as the ARISE I consensus on chronic subdural hematoma management [3].

Over the past several years, multiple randomized trials and systematic reviews have evaluated the role of middle meningeal artery embolization in both surgical and nonsurgical treatment pathways. A large randomized controlled trial demonstrated that embolization as an adjunct to standard management significantly reduced treatment failure compared with standard treatment alone, reinforcing its safety and efficacy in selected patients [1]. Subsequent meta analyses and systematic reviews of randomized trials have further supported these findings, reporting reductions in recurrence and progression while emphasizing heterogeneity in patient selection and treatment paradigms [2,4,5,6]. These data have contributed to a paradigm shift in which embolization is increasingly incorporated into multidisciplinary decision making for chronic subdural hematoma, particularly in neurologically stable patients and those at elevated surgical risk.

Despite this expanding evidence base, a key unresolved clinical question is whether standalone middle meningeal artery embolization can be considered in patients who demonstrate radiological mass effect or herniation features but remain neurologically stable. In current practice, these imaging features frequently prompt surgical evacuation due to concern for abrupt deterioration. Reports describing primary embolization without surgical evacuation exist but remain limited, particularly in cases demonstrating imaging features of cerebral herniation [7]. This report describes a clinically stable adult with chronic subdural hematoma and radiological signs of subfalcine herniation who underwent middle meningeal artery embolization as the sole intervention and achieved sustained clinical recovery with near-complete radiological resolution at eight-month follow-up. This case report has been reported in line with the SCARE 2025 checklist [8].

It is important to emphasize that surgical evacuation remains the standard of care in patients with chronic subdural hematoma presenting with significant mass effect or radiological herniation features. The present report does not seek to challenge established treatment paradigms nor propose a change in clinical practice, but rather to describe an isolated clinical scenario that may contribute to hypothesis generation and future investigation.

## 2. Case Illustration

A 51-year-old male presented with a three-week history of progressively worsening headache, intermittent confusion, and subtle left-sided clumsiness reported by family members. There was no history of recent head trauma, anticoagulant or antiplatelet therapy, alcohol abuse, or known coagulopathy. On presentation, the patient was fully alert with a Glasgow Coma Scale (GCS) score of 15. Neurological examination revealed mild left upper extremity weakness graded 4 plus out of 5 without aphasia, visual field disturbance, or cranial nerve deficit. Vital signs were stable and there were no clinical features of raised intracranial pressure.

**Figure 1:**
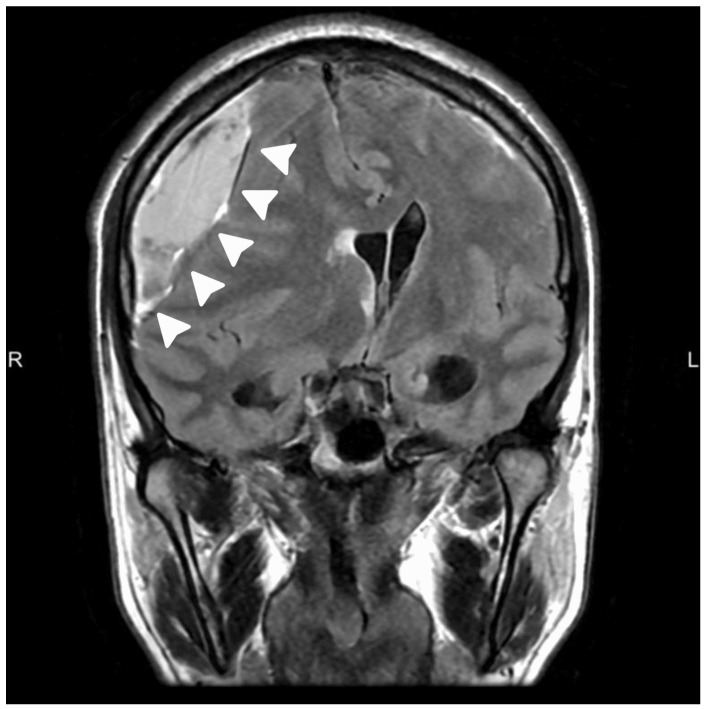
Pre-treatment coronal T2 FLAIR MRI showing a right-sided chronic subdural hematoma (arrow) with significant mass effect and subfalcine herniation.

Magnetic resonance imaging (MRI) of the brain demonstrated a right-sided chronic subdural hematoma with mixed signal intensity and internal septations on coronal T2 FLAIR sequences, causing marked cortical compression and radiological features of subfalcine herniation, as seen in Figure 1. Despite the degree of mass effect, there was no diffusion restriction or parenchymal injury, and the patient remained clinically stable.

Management options were discussed in a multidisciplinary conference involving neurosurgery and neurointervention. Given the marked mass effect, radiological subfalcine herniation, and mild hemiparesis, surgical evacuation was presented as the standard of care and was recommended. The patient remained fully alert without signs of acute deterioration, and the neurological deficit was mild and nonprogressive. After detailed shared decision making with the patient and family, including discussion of potential risks, benefits, and the need for urgent surgical intervention if clinical worsening occurred, a decision was made to proceed with middle meningeal artery embolization as standalone therapy with close clinical and radiological monitoring and immediate surgical backup.

**Figure 2:**
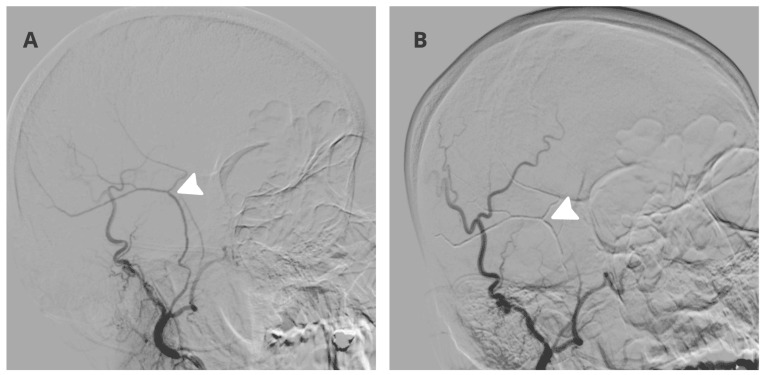
Selective right middle meningeal artery angiography (arrow) demonstrating pathological neovascular supply to the chronic subdural hematoma before embolization (**A**) with complete obliteration (arrow) of the abnormal meningeal branches following embolization (**B**).

The procedure was performed under local anesthesia with mild conscious sedation. Right radial artery access was obtained using a 5 French Glidesheath Terumo Japan. A Simmons 2 diagnostic catheter Radiofocus Terumo Japan was navigated into the right external carotid artery. Selective angiography demonstrated hypertrophy and pathological neovascular blush arising from the right middle meningeal artery consistent with vascularized subdural membranes, as demonstrated in Figure 2A. Distal catheterization of the frontal and parietal branches was achieved using a Vasco 10 microcatheter Balt France over a hybrid microwire Balt France. Superselective angiography confirmed the absence of dangerous anastomoses to the ophthalmic artery or intracranial circulation.

Embolization was performed using a 20 percent mixture of N butyl cyanoacrylate and lipiodol under continuous fluoroscopic guidance. Controlled penetration into the distal dural branches supplying the hematoma membranes was achieved with adequate casting and reflux control. Final angiography demonstrated complete elimination of the pathological meningeal blush with preservation of normal external carotid artery branches, as seen in Figure 2B. No intraprocedural complications occurred and the patient remained neurologically unchanged throughout the intervention.

The post procedural course was uneventful. The patient was monitored after the procedure with serial neurological examinations and routine hemodynamic observation. The patient was managed in an intensive care unit for close observation before transfer to a standard ward. The patient was discharged on the second post procedural day without corticosteroids or antithrombotic therapy. No additional interval neuroimaging was performed between hospital discharge and the eight-month follow-up computed tomography. Clinical monitoring was conducted through scheduled outpatient visits, and repeat imaging was reserved for any clinical deterioration, which did not occur. Serial outpatient clinical evaluations demonstrated progressive resolution of headache and complete recovery of motor strength within four weeks.

**Figure 3:**
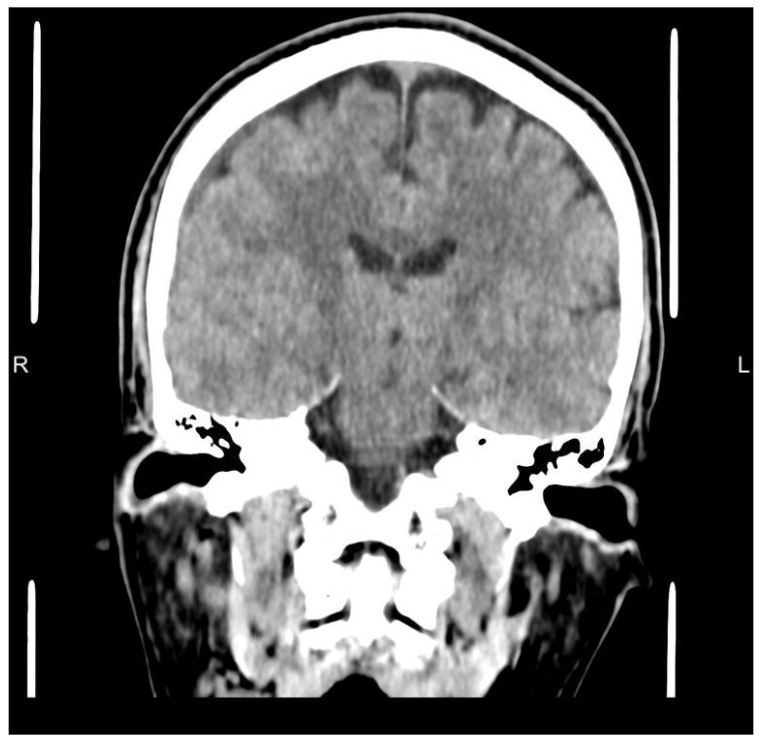
Follow-up non-contrast CT scan at eight months demonstrating near-complete resolution of the chronic subdural hematoma with normalization of midline structures.

Follow-up non-contrast head computed tomography performed eight months after the procedure demonstrated near-complete resolution of the chronic subdural hematoma with restoration of normal ventricular configuration and resolution of midline shift, as shown in Figure 3. The patient remained clinically well without recurrence or need for surgical evacuation.

## 3. Discussion

This case illustrates that, in this specific patient, radiological severity did not translate into immediate neurological deterioration. However, this observation should not be interpreted as evidence that surgical evacuation can be safely deferred in similar cases, as established management guidelines continue to support surgery in the presence of significant mass effect or herniation. The preserved neurological examination despite marked mass effect suggests that chronic hematomas may allow sufficient intracranial compensation, particularly in the absence of acute hemorrhagic components or rapid clinical progression. In such circumstances, addressing the biological drivers of hematoma persistence rather than performing mechanical decompression may represent a rational alternative therapeutic strategy. In this context, standalone embolization may be viewed as a less invasive strategy in selected stable patients, with the theoretical potential to reduce risks associated with surgical evacuation such as infection, anesthesia-related complications, and recurrence, while recognizing that this remains investigational in the setting of significant mass effect.

The successful use of middle meningeal artery embolization as sole therapy in this case supports the hypothesis that elimination of pathological dural vascular supply can permit gradual hematoma resorption even when midline shift and herniation features are present. In conventional practice, significant midline shift and radiological herniation are widely regarded as strong indications for surgical evacuation due to the perceived risk of abrupt neurological decline. Any deviation from this standard approach should be reserved for carefully selected clinically stable patients with immediate access to surgical backup and close monitoring. Importantly, embolization does not provide immediate decompression and therefore does not replace the rapid reduction in mass effect achieved by surgical evacuation. For patients with progressive neurological deficit, reduced level of consciousness, or clinical signs suggesting impending deterioration, surgical evacuation remains the preferred first-line therapy. In the present case, the decision to pursue embolization alone was based on preserved consciousness, a mild and nonprogressive focal deficit, patient preference, and the availability of intensive observation with readiness to proceed to urgent surgical evacuation if deterioration occurred. This clinical context should be considered essential for safety replication. Compared with surgical evacuation, embolization offers the advantage of avoiding general anesthesia, craniotomy or burr hole-related wound complications, and rapid fluid shifts that may contribute to recurrence. However, embolization does not provide immediate decompression and relies on gradual hematoma resorption, which may be inappropriate in unstable patients or those with progressive neurological deterioration. Surgical evacuation remains superior when urgent mass effect reduction is required, whereas embolization may be considered in clinically stable patients in whom close monitoring and rapid surgical conversion are available.

Based on the available evidence and the present case, standalone embolization may be considered in patients with preserved level of consciousness, mild and nonprogressive focal deficits, absence of acute hemorrhagic components on imaging, and availability of intensive clinical monitoring with immediate access to surgical intervention. Conversely, patients with rapid neurological decline, decreased consciousness, or radiological evidence of impending transtentorial herniation should continue to undergo urgent surgical evacuation.

While embolization has been extensively studied as an adjunct to surgery or conservative management, data specifically addressing embolization as definitive therapy remain limited. Randomized trials have demonstrated reductions in treatment failure when embolization is incorporated into standard care, but these studies largely excluded patients with significant mass effect or mandated surgical intervention based on imaging criteria alone [1]. Meta analyses of randomized and observational studies similarly emphasize benefit while highlighting substantial heterogeneity in inclusion criteria and treatment pathways [2,4,5].

From a clinical decision making standpoint, this case reinforces the importance of prioritizing neurological status and disease chronicity over imaging findings alone. Expert consensus statements increasingly acknowledge that chronic subdural hematoma management should be individualized, incorporating patient stability, trajectory, and access to close monitoring, rather than applying uniform surgical thresholds based solely on radiological measurements [3]. Prior case reports and small series have described primary embolization in selected patients, generally involving individuals without marked radiological herniation or with contraindications to surgery. In contrast, the present case involved significant midline shift and mild hemiparesis, yet preserved consciousness and stable neurological status. This distinction highlights that radiological severity alone may not uniformly predict clinical instability, and careful individualized assessment remains central to decision making [7,9].

Technical execution is particularly critical when embolization is pursued as definitive therapy. Complete devascularization of the dural membranes is necessary to suppress ongoing angiogenesis and inflammatory exudation, while meticulous angiographic evaluation is required to avoid non-target embolization. Potential complications of middle meningeal artery embolization include non-target embolization leading to ischemic stroke, cranial nerve palsy due to dangerous anastomoses, scalp or skin necrosis, seizure, post procedural headache, and contrast-related adverse events. Although reported complication rates in contemporary series are low, these risks must be carefully weighed against the anticipated benefit, particularly when embolization is considered as standalone therapy in patients who would otherwise be surgical candidates [10]. The absence of complications in this case likely reflects both favorable vascular anatomy and adherence to strict superselective embolization principles.

Several limitations of this case must be acknowledged. First, this is a single patient experience and therefore cannot be generalized to all patients with chronic subdural hematoma and herniation features. Second, the patient was neurologically stable with preserved consciousness, a condition that may not apply to patients with fluctuating mental status or rapid deterioration. Third, management was undertaken in a center with dedicated neurointerventional expertise and the ability to provide close clinical follow-up, which may limit applicability in other settings. Fourth, the absence of intermediate imaging limits characterization of the temporal pattern of hematoma resolution. Finally, although the eight-month follow-up demonstrates durable radiological and clinical resolution, longer term outcomes beyond this period remain unknown. Late recurrence, delayed adverse events, or other complications beyond the available follow-up window cannot be excluded. Longer-term follow-up is required to better define durability and late safety after standalone embolization in this clinical context.

Importantly, individual clinical decisions made in highly controlled circumstances cannot be extrapolated to broader patient populations. A single case report cannot provide sufficient scientific basis to alter standard management strategies, and any deviation from established surgical indications should be undertaken only within rigorous clinical governance frameworks and with immediate access to surgical intervention. This report is therefore intended solely to contribute to academic discussion and hypothesis generation rather than to inform routine clinical decision making.

## 4. Conclusions

This case illustrates a potential treatment strategy in a highly selected clinically stable patient and should not be interpreted as evidence to support routine standalone embolization in all patients with radiological herniation features. The findings are hypothesis generating and underscore the need for prospective studies and larger case series before broader clinical adoption can be considered.

Under carefully controlled conditions and with close clinical monitoring, primary embolization in this context should be regarded as an exceptional, exploratory approach rather than a recommended therapeutic strategy. However, this approach cannot be recommended as standard practice on the basis of a single case and requires validation in larger prospective studies.

## Data Availability

All data generated or analyzed during the study are included in this published article.
